# Type 2 Diabetes and Cognitive Dysfunction: Cardiorespiratory Fitness and Physical Activity Matters

**DOI:** 10.1155/jdr/1311227

**Published:** 2026-08-02

**Authors:** Sandra Rojas Vega, Andrea Solera-Herrera, Ramin Vafa, Daniel Acero-Moreno, Veronika Wahrmann, Lukas Scheef

**Affiliations:** ^1^ Institute of Movement Neuroscience, German Sport University Cologne, Cologne, Germany, dshs-koeln.de; ^2^ School of Physical Education and Sports, University of Costa Rica, San Pedro, Costa Rica, ucr.ac.cr; ^3^ Children′s Hospital Amsterdam, Cologne, Germany; ^4^ Department of Internal Medicine, Augustinerinnen Hospital, Cologne, Germany; ^5^ Biomedical Engineering in Sports Medicine, Department for Mathematics, Informatics, and Technology, University of Applied Sciences, Koblenz, Germany, fhwn.ac.at

**Keywords:** cardiorespiratory fitness, CODEX, cognition, diabetes, physical activity

## Abstract

**Background:**

Diabetes is associated with an increased risk of cognitive dysfunction. We are aimed at examining cognitive performance in people with Type 2 diabetes mellitus (T2DM) and their correlation with physical activity (PA)/fitness level and mood.

**Methods:**

A cross‐sectional study involving 47 individuals with T2DM, 30 diabetes‐free individuals (controls): ages of 40–65 years and without dementia. Subjects were evaluated using a neuropsychological test battery that included Stroop, VLMT, CORSI, TMT and neuropsychiatric scales. The Global Physical Activity Questionnaire (GPAQ) was assessed. Physical fitness was evaluated using a maximal cardiopulmonary exercise testing (CPX).

**Results:**

Compared with controls, people with T2DM had lower cardiorespiratory fitness (CRF) and more frequent cognitive performance below 1 SD of the normative value. Those individuals with T2DM, who displayed low fitness, showed significantly poorer cognitive inhibition (Stroop, *p* < 0.001), processing speed (TMT‐A, *p* < 0.001), cognitive flexibility (TMT‐B, *p* < 0.001), visuospatial memory (CORSI, *p* < 0.001) and memory consolidation (VLMT, *p* < 0.001). Higher ventilatory and metabolic CRF markers and higher PA levels correlated significantly with higher cognitive performance. None of the neuropsychological or psychiatric background variables correlated significantly with any of the cognitive scales.

**Conclusions:**

Individual CRF markers and PA levels correlated positively with cognitive performance in people with T2DM and in nondiabetic individuals. However, individuals with T2DM showed at least two to three times more frequent cognitive performances more than 1 SD below norm as compared with controls. Among people with T2DM, those with low aerobic fitness/PA levels showed significantly lower cognitive performance, especially in the attention‐concentration, executive functioning, episodic memory and visuospatial processing domains.

## 1. Introduction

Individuals with Type 2 diabetes mellitus (T2DM) may often develop cognitive dysfunction and mood impairments as comorbidities [1]. The frequency and severity of cognitive dysfunction are closely associated with longer diabetes duration, older age and the presence of diabetes‐related comorbidities. Cognitive dysfunction varies widely in type and severity and can range from mild cognitive impairment (MCI) to dementia (vascular and/or neurodegenerative genesis). Changes in cognitive performance in MCI involve one or more cognitive domains (e.g., memory, processing speed and executive function), typically performed 1.5 SDs below the normative data. In MCI, this reduced cognitive performance is accompanied by subjective memory complaints and nonmemory‐related cognitive impairments, although activities of daily living remain largely intact [2]. For some individuals, performance about 1 SD below average can still fall within the range of normal variation among cognitively healthy people; however, detecting such subtle decrements may be important in populations at risk for cognitive decline. In diabetes, these subtle cognitive changes often emerge early and can progress gradually over time [3], making their identification clinically informative. Detecting these early changes also provides an opportunity to encourage optimal disease management, including regular physical activity (PA), which is known to support cognitive health [4].

Several cross‐sectional studies have assessed cognitive function in people with diabetes; most of them, conducted in older adults, use a single or a limited neuropsychological test battery. Systematic reviews of these studies [5, 6] conclude that people with T2DM perform worse in many domains than those without diabetes. Cognitive dysfunction may reduce patient compliance and adversely impact self‐care management and glycemic control. As a key component of a healthy lifestyle, PA can favourably influence several cardiometabolic risk factors associated with cognitive dysfunction, including hypertension, insulin resistance and dyslipidemia. Exercise training improves cardiorespiratory fitness (CRF) and ameliorates metabolic markers [7].

In clinical medicine, aerobic exercise is often used as an intervention to promote general health and to obtain both physical and mental benefits. Chronic endurance exercise has been shown to induce neuroplastic changes in brain areas that regulate learning and memory, improving cognitive health [8, 9]. Based on epidemiological/clinical studies in healthy subjects, obese adults, individuals with cardiac failure, as well as older people, showing that PA is associated with improvements in cognitive performance, Srikanth et al. [10] recommended, among others, physical exercise for the effective management of cognitive dysfunction by people with T2DM. Recent meta‐analyses of longitudinal exercise interventions highlight that PA and its effects on CRF play a crucial role in preserving and improving cognitive function (assessed by the Mini‐Mental State Examination and/or the Montreal Cognitive Assessment) in people with T2DM [11, 12].

Although weekly PA levels may influence the aerobic capacity of patients with T2DM, there is a lack of studies that meticulously quantify individual PA, rigorously assess markers of both submaximal and maximal CRF (ventilatory and metabolic) and simultaneously evaluate cognitive performance using a battery of neuropsychological tests in middle‐aged adults with diabetes.

In the present study, we assess cognition, fitness and PA multidimensionally, rigorously controlling for confounding variables. Using objective (CPX testing) and subjective (Global Physical Activity Questionnaire [GPAQ]) measures of fitness and activity, alongside a detailed neuropsychological battery, allows for nuanced analysis of specific cognitive domains.

We hypothesised that (a) higher CRF markers by individuals with T2DM correlate positively with cognitive performance, and (b) the amount of PA is positively linked with cognitive performance. To our knowledge, this is the first comprehensive exploratory study to examine cognitive performance in individuals with T2DM in relation to individual CRF markers and overall PA.

## 2. Methods

### 2.1. Study Population

This exploratory cross‐sectional study involved initially 137 middle‐aged volunteers (40–65 years). Participants were recruited through newspaper announcements and/or contact with diabetologists, endocrinologists and community clinics in Cologne and Bonn (Germany) that offer clinical training programmes on diabetes. The recruitment duration was 18 months. Data for the present analysis were obtained from the CODEX study (Cognition, Diabetes, and Exercise), a larger study that included cardiopulmonary exercise testing (CPX), neuropsychological test screening and a comprehensive battery of structural and functional MRI protocols (to be reported elsewhere). For an overview of the methods and variables in this study, see Table 1. Initially, an interview was performed to evaluate the following exclusion criteria: acute or chronic severe neurological disease, current and/or past history of psychiatric illness, taking medication that may influence cognitive performance and contraindication to perform a CPX.

**Table 1:** Overview of methods and main targeted variables assessed in the CODEX Study.

Target	Method	Parameter
Cardiorespiratory fitness	Spiroergometry	Oxygen consumption at 4 mmol/L Lactate (*V̇*O_2_4La) Power at 4 mmol/L La (W), *V̇*O_2_peak (ml.kg^−1^ min^−1^)

Cognition	Stroop test, VLMT, TMT A‐B, CORSI, GVT	Raw scores, percentile rank scores

Mood assessment	BDI, STAI, PANAS	Scores

Physical activity (PA) level	Global Physical Activity Questionnaire: GPAQ	Total PA (METS.min. wk^−1^) sedentary time (h.d^−1^)

Neurotrophic factors	Blood sample (ELISA)	Concentration in serum

MRI	3 T MRI, DTI voxel	Cortex volumen, gray matter density Hippocampus volume (cm^3^)

Diabetes	Blood sample	Glycemia (mg/dL), HBA_1c_ (%), cholesterol, triglyceride

**Figure 1:**
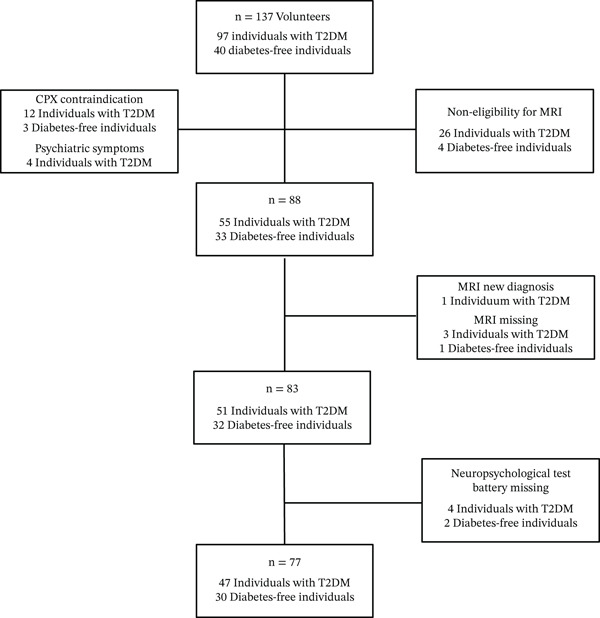
The flow chart shows the number of participants and the reasons for dropouts and exclusion throughout the recruitment process.

Additionally, subjects were excluded based on eligibility for an MRI scan. After the subject′s MRI analysis, individuals were excluded because of major abnormalities on brain scans, such as cerebral infarction or tumour. Seventy seven volunteers met the study inclusion criteria: 47 individuals with T2DM and 30 diabetes‐free individuals. The flowchart of the study is indicated in Figure 1. All participants signed an informed consent at each clinical and cognitive examination session of the study. Participants′ characteristics were obtained from medical evaluation and a set of questionnaires (sociodemographic, GPAQ and German vocabulary test [GVT]). Participants were evaluated in three sessions, each lasting 2 h, to avoid fatigue. Before each session, the blood glucose level was determined to exclude hypoglycemia. The study was conducted in accordance with the principles of the Declaration of Helsinki. It was approved by the local ethics committees of the German Sport University and the Rheinische Friedrich‐Wilhelm University Bonn, with the Ethical Approval Numbers 050/14 and 229/14, respectively.

### 2.2. CPX

At the first session, participants underwent a medical evaluation including medical history, physical examination, resting ECG, blood measures and an incremental CPX with continuous ECG and ventilatory measurements until volitional exhaustion. Blood pressure was recorded at rest before CPX and at every 25‐W stage of CPX. Capillary blood samples were taken within the last 30 s of each stage of the test. Capillary blood was analysed using a clinical device to determine the lactate concentration (EKF Diagnostic, BIOSEN C‐Line, Barleben, Germany), yielding an individual blood lactate profile curve. Reported CRF markers were determined by measuring oxygen consumption (*V̇*O_2_) at the metabolic aerobic–anaerobic threshold of 4‐mmol/L lactate (4La): *V̇*O_2_4La and *V̇*O_2_peak during CPX. An additional CRF marker was determined individually from a plot of blood lactate concentration (4 mmol/L) against power in Watts (Pow4La). *V̇*O_2_4La was calculated as a reasonably accurate submaximal predictor of aerobic fitness, as some participants did not perform to maximal exhaustion. Since better outcomes of submaximal cardiopulmonary fitness are reflected by a higher *V̇*O_2_4La, with a typically lower heart rate (HR) at a given work rate [13], this procedure ensured comparability of relative exercise intensities across all participants [14, 15].

### 2.3. Cognitive Assessment

The neuropsychological test battery of standardised tests with available normative data (Table 2) was assessed using computer‐based methods and paper‐and‐pencil tests. Paper‐and‐pencil tests comprised the verbal learning memory test (VLMT) [16] and the GVT [17]. The computerised neuropsychological battery, Vienna Test System (Schuhfried GmbH, Moedling, Austria), was administered using the following tests: Stroop test (S7 form), The Trail Making Test (TMT) (S1 form) and CORSI block tapping test (forward, S1 form). The normative data for the computerised tests Stroop, TMT and CORSI were obtained from the Dr. Schuhfried Company′s research laboratory in Vienna (Austria) and from the University of Bonn (Germany) for VLMT.

**Table 2:** Representative norm sample considered for each test. EU educational level: no school‐leaving qualification; EU educational level 2: compulsory schooling or an intermediate secondary school but without completing vocational training; EU educational level 3: completed vocational training or a course at a technical college; EU educational level 4: school‐leaving qualification at university entrance level; EU educational level 5: university degree.

Test	*n*	Gender	Age	Education
Stroop	270	*f* = 146	44.03 ± 16.37	1: educational level 1
				45: educational level 2
Test form S7		*m* = 121		122: educational level 3
			(Range: 15–80)	77: educational level 4
				25: educational level 5

CORSI	295	*f* = 295	45.52 ± 16.84	1: educational level 1
				45: educational level 2
				122: educational level 3
Test form S1		*m* = 272	(Range: 16–85)	77: educational level 4
				25: educational level 5

TMT	600	*f* = 307	Mean = 44.84	3: educational level 1
				75: educational level 2
			SD = 16.57	236: educational level 3
Test form S1		*m* = 293	(Range: 16–83)	204: educational level 4
				82: educational level 5

VLTM	515	*f* = 198	Group 1: 7.74 ± 1.07	Group 3 (15–29 years), 4 (30–49 years) and 5 (> 50 years) all included education level 3, 4 and 5
			Group 2: 12.6 ± 1.78	
			Group 3: 22.37 ± 3.93	
		*m* = 317	Group 4: 37.5 ± 6.53	
			Group 5: 60.64 ± 8.98	
			(Range: 6–79)	

**Figure 2:**
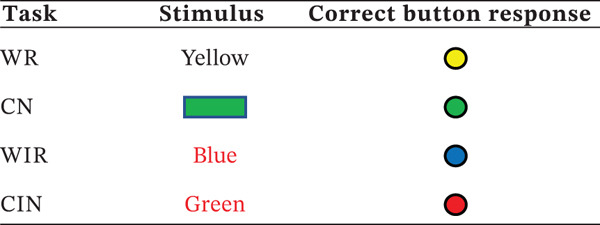
Response matrix used in the Stroop task. The different tasks, along with their corresponding stimuli, are displayed. Abbreviations: WR, word reading; CN, colour naming; WIR, word interference reading; CIN, Colour interference naming.

The Stroop test evaluates executive function, inhibitory control, cognitive flexibility and attention. The test itself is divided into four subtasks (Figure 2). For Tasks 1 (word reading [WR]) and 2 (colour naming [CN]), reaction time and errors while reading and naming one of four colours were measured, providing baseline values. Task 3 (word interference reading [WIR]) and 4 (colour interference naming [CIN]) impose interferences between the colour and the written word by using different font colours. Effects of interference show up in reaction speed, with a longer time to read/name once interference between the word and its print colour is introduced. Data were analysed using the difference scores between baseline and interference conditions. The Stroop test is reported as the interference tendency; a higher tendency indicates worse function. Based on how strongly the interference condition influenced the subject′s reaction time, we allocated participants to two performance categories. *Impaired* demarcates performances above expected norm values, a massive increasing interference tendency, whereas *normal* refers to a performance with a stable interference tendency.

During the VLMT, subjects must repeat an unstructured learning list of 15 words in five trials. Following a second list in one trial (distraction list B, 15 words) is shown with immediate recall. After 30 min, the participants are asked to repeat the learning list A (delayed recall). In the final step, a list of 50 words is read aloud. With the help of this recognition list and yes/no answers, a subject is asked which words from list A the subject can recognise. The main variables of the VLMT are *learning performance* (sum of correct responses after 5 learning trials), *consolidation* (correct responses following distraction) and *retrieval* (correctly recognised words out of 50 alternatives).

The TMT consists of two parts. In Part A, subjects are instructed to connect numbered circles in ascending order as quickly as possible. In Part B, subjects have to connect letters or digits alternately. The main target variables are the times needed for Parts A and B, with a longer time indicating worse performance in cognitive processing speed in Part A, and in cognitive flexibility, working memory in Part B. Performance in TMT makes demands on different neuropsychological domains: attention, visuomotor processing speed and executive function, such as cognitive flexibility and working memory.

In the CORSI test of immediate block span, nine irregularly distributed cubes are displayed on the monitor. A pointer in the form of a hand ‘taps’ on a given number of cubes one after the other. The participant has to tap subsequently in the order shown. The test is performed in an ascending sequence of length. After three items, the number of cubes increases by one, continuing until an 8‐item sequence. The test is terminated if the subject answers three consecutive items incorrectly. By increasing sequence length, the capacity of spatial working memory can be measured. The main target variable is the immediate block span: The longest block sequence correctly reproduced at least twice.

### 2.4. Neuropsychiatric Questionnaires

To rule out the possible effect of neuropsychiatric symptoms causing cognitive dysfunction, psychiatric questionnaires (anxiety and depression) were administered at the third session and evaluated as covariates. The questionnaires were applied in the following order: the Beck Depression Inventory (BDI) [18] German Version [19], the Positive and Negative Affect Scale (PANAS) [20] and the State‐Trait Anxiety Inventory (STAI) [21]. The PANAS questionnaire contains 10 positive affect‐related adjectives and 10 negative affect‐related adjectives to measure positive and negative affect, respectively. Participants rated each adjective according to what they felt from 1 (*not at all*–*slightly*) to 5 (*extremely*). STAI contains 40 items, with 20 for trait anxiety and 20 for state anxiety. STAI scores range from 20 to 80 (20 = *not afraid* and 80 = *maximum intensity of anxiety*). BDI is aimed at measuring depressive mood symptoms with 21 multiple‐choice items; scores range between 0 and 63. A BDI score lower than 11 was considered normal, a score between 11 and 17 was considered mild to moderate depressive symptoms, and a BDI ≥ 19 or higher was regarded as moderate or severe depression [19].

### 2.5. PA and Sedentary Time Assessment: GPAQ

PA level was calculated based on self‐reporting of the global GPAQ of the WHO [22]. The GPAQ included questions on intensity and frequency of PA. It assesses three domains in which PA is performed (occupational PA, transport‐related PA and PA during leisure time). The total amount as the PA (PA total) is defined by total time in minutes per week (min. wk^−1^) spent physically active, calculated in METs. A value of 4 METs was assigned to moderate intensity, and 8 METs to vigorous intensity. To improve the accuracy of the survey, all participants were interviewed about their self‐reported time being physically active to assign the recalled activities the appropriate intensity levels. Following Chu et al. [23] participants were classified as insufficiently active when the subject did not meet one of the following recommendations:

1. At least 30 min of moderate intensity activity or walking per day on at least 5 days of a typical week,
2. 20 min of vigorous‐intensity activity per day on at least 3 days of a typical week or
3. 5 days of any combination of walking and moderate or vigorous‐intensity activities achieving a minimum of at least 600 METS‐min per week

To calculate sedentary time, participants were asked about the time spent sitting each day.

### 2.6. Statistical Analysis

Statistical analyses were performed using SPSS 23 (IBM Corp., Armonk, New York, United States). Descriptive data are shown as mean ± standard deviation. Statistically significant results were reported at *p* values < 0.05. Independent Student′s *t*‐tests were used to compare physiological measures, cognitive scores and neuropsychiatric scales between two groups (individuals with T2DM and controls).

Cognitive status was considered *impaired* when the mean score on each test was < 1 SD below the age‐ and gender‐matched normative reference data. In addition, we calculate the percentage of ‘impairment’ for each cognitive test within each group.

The effect size was estimated with Cohen′s *d* for two means comparisons. The power of 0.80 was calculated using the following values: Group 1 (control group) *n* = 30 and Group 2 (people with T2DM) *n* = 47, with an effect size > 0.66 and *α* = 0.05.

To examine overall associations between cognitive test performance and markers of CRF/PA, Pearson correlation analyses were conducted on the total sample (*n* = 77) and within the T2DM group (*n* = 47).

## 3. Results

### 3.1. Comparison Between Individuals With T2DM and Controls

#### 3.1.1. Demographic and Physiological Variables

Participants′ characteristics are summarised in Table 3. Independent *t*‐tests revealed significant group differences for waist circumference, BMI, HbA_1c_, cholesterol, triglycerides and blood glucose. Both groups differed significantly in age; however, the difference was below 5 years. No significant group differences were observed for educational level, verbal intelligence quotient (estimated by GVT), resting systolic or diastolic blood pressure, or level of PA.

**Table 3:** Demographics and physiological variables of individuals with diabetes and control subjects. Plus–minus values are means and standard deviations.

Variable	Individuals with Diabetes (*n* = 47)	Controls (*n* = 30)	*p* value
Gender (f/m)	14/33	15/15	—
Age (years)	57.6 ± 6	54 ± 8	0.02
Waist circumference (cm)	105.7 ± 14.7	98.4 ± 7.6	0.013
BMI (kg/m^2^)	30.4 ± 4.9	26.7 ± 3.4	0.01
HbA_1c_ (%)	7.43 ± 1.5	5.5 ± 0.4	< 0.001
Glycemia (mg/dL)	163 ± 49	92.6 ± 15	< 0.001
Cholesterol	189 ± 42	210 ± 52	0.061
Triglycerides	215 ± 158	150 ± 121	0.061
Education level (1–5)	3.8 ± 1.0	3.9 ± 0.9	NS
Diabetes duration	8.2 ± 6.2	NA	—

Abbreviation: NA, not applicable.

#### 3.1.2. CPX

There were significant differences between people with T2DM and controls for the following variables: HR at rest, at 4La and HRmax. As shown in Table4, the fitness markers Power at 4La, *V̇*O_2_4La and *V̇*O_2_peak were significantly higher in individuals without diabetes than in those with T2DM. During the exercise period, the maximum achieved blood pressure (systolic or diastolic) did not differ significantly between the two groups examined.

**Table 4:** Physiological responses in CPX of all participants.

	Individuals with Type 2 diabetes (*N* = 47)	Controls (*N* = 30)	*p* value
HR rest (bpm)	79.9 ± 8.3	69.3 ± 8.7	0.01 ^∗^
HR at 4La (bpm)	127 ± 18	137 ± 12	0.014 ^∗^
HR max(bpm)	150 ± 15.6	160 ± 14.5	0.005 ^∗∗^
Resting BP (mmHg)	131/84 ± 17/8	129/85 ± 16/9	NS
Maximal BP (mmHg)	216/86 ± 31/12	205/84 ± 29/10	NS
La at rest (mmol/L)	1.07 ± 0.6	1.1 ± 0.5	NS
La max (mmol/L)	6.2 ± 1.7	6.8 ± 1.5	NS
Pow 4La (W/kg)	1.29 ± 0.4	1.55 ± 0.4	0.011 ^∗^
*V̇*O_2_4La (ml.kg^−1^ min^−1^)	19.2 ± 6.2	23.2 ± 5.5	0.005 ^∗∗^
*V̇*O_2_peak (ml.kg^−1^ min^−1^)	24.6 ± 6.6	28.6 ± 6.5	0.01 ^∗^

*Note:* Values are means ± SD. Pow 4La: power at 4 mmol lactate. *V̇*O_2_4La: oxygen consumption at 4 mmol Lactate. *V̇*O_2peak_: peak oxygen consumption.

Abbreviations: HR, heart rate; La, lactate.

^∗^Significant *p* < 0.05,  ^∗∗^high significant *p* < 0.01.

#### 3.1.3. PA and Sedentary Time: GPAQ

##### 3.1.3.1. Total PA

There were no significant differences in PA 602.6 ± 366 and 551.7 ± 387 METS^.^min^.^wk^−1^ for individuals with T2DM and controls, respectively.

##### 3.1.3.2. Total Sedentary Time

We found a significant difference (*t* = 2.14; *p* = 0.04) between the two groups. The average sedentary time was subjects with T2DM (8.96 ± 3.5) and diabetes‐free individuals (7.28 ± 3.2 h/day).

#### 3.1.4. Psychiatric Questionnaires

None of the participants showed results indicating psychiatric disease. STAI Trait scores showed significant differences between individuals with T2DM (34.5 ± 9) and diabetes‐free controls (40.1 ± 9; *t* = −2.79, *p* = 0.007).

#### 3.1.5. Cognition

No between‐groups differences were observed in neuropsychological test scores (Table 5).

**Table 5:** Cognitive performance in neuropsychological tests of all participants. Values are means ± SD, 95% confidence intervals for cognitive outcomes (CI95%).

	Individuals with T2DM		Controls			
	*n* = 47		*n* = 30			
	*M* *e* *a* *n* *s* ± *S* *D*	CI 95%	*M* *e* *a* *n* *s* ± *S* *D*	CI 95%	*t*‐value	*p* value
WIR tendency	0.14 ± 0.09	0.12–0.16	0.13 ± 0.07	0.01–0.15	0.849	0.398
CIN tendency	0.13 ± 0.09	0.10–0.15	0.12 ± 0.13	0.07–0.17	0.267	0.790
VLMT learning	47.1 ± 8.35	44.6–49.5	48.3 ± 8.28	45.2–51.4	−0.653	0.516
VLMT consolidation	1.77 ± 2.39	1.06–2.47	1.57 ± 1.79	0.90–2.24	0.392	0.697
VLMT retrieval	13.1 ± 2.22	12.5–13.58	12.6 ± 3.00	11.4–13.7	0.941	0.350
TMT A	25.3 ± 5.43	23.7–26.9	24.9 ± 5.01	23.1–26.8	0.300	0.765
TMT B	36.9 ± 13.3	33.0–40.8	33.2 ± 9.07	29.8–36.6	1.341	0.184
CORSI USB	4.83 ± 0.87	4.58–5.08	4.73 ± 0.64	4.49–4.97	0.524	0.602

A diminished performance in several cognitive tasks was at least two to three times more frequent in individuals with T2DM than in healthy controls, including executive function (Stroop: interference tendency), delayed free recall (VLMT: learning and consolidation), cognitive flexibility and working memory (TMT‐B) and spatial working memory (CORSI). (Table 6).

**Table 6:** Individuals with Type 2 diabetes and diabetes‐free individuals (controls) are characterised based on their performance on the neuropsychological assessment.

	Raw test scores		Statistic values	Raw test scores		Statistic values
	Individuals with T2DM			Controls		
	Normal	Impaired		Normal	Impaired	
**Stroop** ^∗^						
WIR tendency (s)	0.11 ± 0.06 (*n* = 36)	0.25 ± 0.10 (*n* = 11) 23.4%	*t* = 5.62, **p** < 0.001**,**ES = −1.93	0.11 ± 0.05 (*n* = 27)	0.27 ± 0.04 (*n* = 3) 10%	*t* = 5.08, **p** < 0.001, ES = −3.12
CIN Tendency (s)	0.10 ± 0.07 (*n* = 32)	0.18 ± 0.13 (*n* = 15) 29.8%	*t* = 2.88, **p** = 0.006, ES = −0.90	0.08 ± 0.07 (*n* = 25)	0.29 ± 0.19 (*n* = 5) 16%	*t* = 4.39, **p** < 0.001, ES = 2.15
**VLMT**						
Learning total	48.03 ± 7.6 (*n* = 44)	32.33 ± 1.5 (*n* = 3) 6.4%	*t* = 3.53, **p** < 0.001, ES = 2.11	49.44 ± 7.8 (*n* = 27)	38.83 ± 5.9 (*n* = 3) 10%	*t* = −2.37, **p** = 0.025, ES = 1.45
Consolidation ^∗^	0.39 ± 1.36 (*n* = 31)	4.44 ± 1.5 (*n* = 16) 34%	*t* = 9.34, **p** < 0.001, ES = 2.88	0.67 ± 1.3 (*n* = 21)	3.7 ± 0.5 (*n* = 9) 30%	*t* = −6.58, **p** < 0.001, ES = −2.62
Retrieval	13.52 ± 1.65 (*n* = 44)	7.33 ± 1.15 (*n* = 3) 6.4%	*t* = −6.36, **p** < 0.001, ES = 3.80	13.4 ± 2.04 (*n* = 26)	7.0 ± 2.16 (*n* = 4) 16%	*t* = 5.82, **p** < 0.001, ES = 3.12
**TMT** ^∗^						
**TMT-A** (s)	22.93 ± 0.72 (*n* = 35)	32.26 ± 5.44 (*n* = 12) 26%	*t* = 7.79, **p** < 0.001, ES = 2.61	22.93 ± 2.8 (*n* = 23)	31.54 ± 5.1 (*n* = 7) 23%	*t* = 5.80, **p** < 0.001, ES = 1.48
**TMT-B** (s)	34.4 ± 9.9 (*n* = 36)	63.8 ± 15.9 (*n* = 11) 23.4%	*t* = 7.06, **p** < 0.001, ES = −2.80	32.01 ± 7.4 (*n* = 28)	49.5 ± 18.1 (*n* = 2) 6.6%	*t* = 2.97, **p** = 0.006, ES = 5.89
**CORSI**						
UBS forwards	5.14 ± 0.7 (*n* = 36)	3.81 ± 0.4 (n = 11) 23.4%	*t* = 7.70, **p** < 0.001, ES = 1.98	4.92 ± 0.5 (*n* = 25)	3.80 ± 0.5 (*n* = 5) 6.6%	*t* = 4.69, **p** < 0.01, ES = −2.3

*Note:* Values are raw scores, means and standard deviations—percentage of individuals within the group with cognitive impairment. Impairment is defined as a performance below 1 SD of normative values (in parentheses, number of individuals).

Abbreviations: CIN, colour interference naming; ES, effect size; WIR, word interference reading.

^∗^A higher value indicates a worse test performance.

#### 3.1.6. Correlations Between Cognition and Fitness Markers or Physiological Variables of All Subjects

Several CRF markers/levels of PA were positively correlated with total learning and retrieval (VLMT) (Table 7). Inverse correlations were observed for CRF markers and cognitive flexibility (TMT‐B). There was a significant correlation between the maximal (systolic only) blood pressure achieved during CPX and the total amount of PA (*r* = 0.295, *p* = 0.009). Individual HbA_1c_ (%) correlates negatively with the amount of time to complete the TMT‐B test (*r* = 0.258, *p* = 0.023).

**Table 7:** Pearson correlation values between cognitive performance and markers of CRF and Total PA in all subjects (*n* = 77).

Variable	Pow4La	*p* value	*V̇*O_2_4La	*p* value	*V̇*O_2peak_	*p* value	PA total	*p* value
Stroop naming interference tendency	*r* = −0.08	0.496	*r* = −0.026	0.82	*r* = 0.025	0.83	*r* = 0.20	0.08
VLMT learning	*r* = 0.264	0.02 ^∗^	*r* = 0.231	0.043 ^∗^	*r* = 0.211	0.66	*r* = 0.16	0.16
VLMT consolidation	*r* = 0.186	0.105	*r* = 0.122	0.29	*r* = 0.18	0.12	*r* = 0.11	0.34
VLMT retrieval	*r* = 0.10	0.38	*r* = 0.052	0.65	*r* = 0.144	0.21	*r* = 0.24	0.04 ^∗^
TMT‐B	*r* = −0.31	0.006 ^∗∗^	*r* = −0.28	0.01 ^∗^	*r* = −0.32	0.004 ^∗∗^	*r* = 0.14	0.23
CORSI	*r* = −0.20	0.86	*r* = −0.098	0.39	*r* = 0.20	0.86	*r* = 0.63	0.58

*Note:* Asterisks indicate statistical significance levels:*p <0.05 denotes significance, and **p < 0.01 denotes high significance.

### 3.2. Comparison Among Individuals With T2DM

#### 3.2.1. Demographic, Anthropometric or Physiologic Variables

In the analysis focused on individuals with T2DM (cognitive subgroups: normal/impaired), there was no significant difference in blood pressure levels (at rest or maximum during CPX), blood glucose levels, cholesterol, triglycerides, age, BMI and diabetes duration.

#### 3.2.2. CPX, PA (GPAQ) and Cognition

Based on the cognitive performance (normal/impaired) in cognitive tests, the Student′s *t*‐test revealed that CRF markers differed significantly between subjects with ‘normal’ and ‘impaired’ cognition (Stroop and VLMT), specifically among individuals with T2DM, as follows:

• Stroop test: CIN task:

*V̇*O_2_4La (ml/kg/min): ‘normal’ (20.3 ± 6.9), ‘impaired’ (16.79 ± 2.9), *t* = −2.44, *p* = 0.02;

Pow4La (Watt/kg): ‘normal’ (1.38 ± 0.47), ‘impaired’ (1.11 ± 0.24), *t* = −2.02, *p* = 0.049;

V̇O_2_peak (mL/kg/min): ‘normal’ (26.26 ± 6.6), ‘impaired’ (20.57 ± 4.62), *t* = −3.36, *p* = 0.002;

PA_total_ (METS^.^min^.^wk^−1^): ‘normal’ (695.6 ± 36), ‘impaired’ (404.0 ± 29), *t* = −2.717, *p* = 0.009.

• VLMT: consolidation

HRmax (bpm): *‘*normal’ (152 ± 6.6), ‘impaired’ (140 ± 4.62), *t* = −3.36, *p* = 0.002.

### 3.2.3. Correlations Between Cognition and Fitness Markers or Physiological Variables of Individuals With T2DM

#### 3.2.3.1. Cognition, CRF Markers and Level of PA

**Figure 3:**
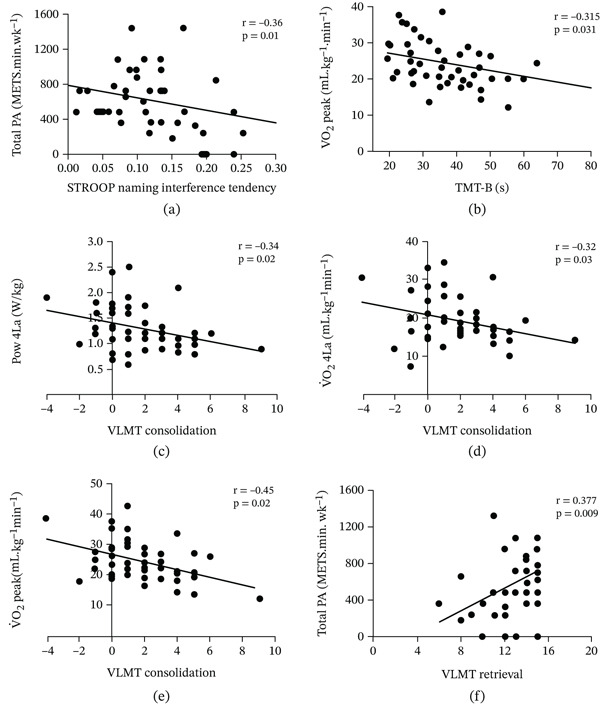
Correlations between (a) amount of physical activity (total) PA in METS. min per week and the naming interference tendency (reaction times in seconds) Stroop test. (b) peak *V̇*O_2_ and working time in Trail Making Test Part B (TMT‐B). (c) Power at 4‐mmol lactate (PoW4La), (d) *V̇*O_2_ at 4‐mmol lactate and (e) peak *V̇*O_2_ in VLMT consolidation. (f) amount of physical activity in METS. min per week and VLMT retrieval. Points data represent subgroups of the diabetic group (*n* = 47).

The analysis focusing on individuals with T2DM (Table 8 and Figure 3) revealed significant correlations between CRF markers and learning consolidation (VLMT). Total PA levels correlated positively with total learning/memory retrieval (VLMT) and negatively with reaction time for CIN (Stroop test). *V̇*O_2_peak showed inverse correlations with processing speed (TMT‐B: cognitive flexibility). An inverse correlation between maximal systolic blood pressure (CPX) and the reaction time for CIN in the Stroop test (*r* = −0.446, *p* = 0.002) was observed, and a positive correlation with the total amount of PA (*r* = 0.376, *p* = 0.009).

**Table 8:** Pearson correlation between cognitive performance and markers of CRF and Total PA by individuals with Type 2 diabetes (*n* = 47).

Variable	Pow4La	*p* value	*V̇*O_2_4La	*p* value	*V̇*O_2peak_	*p* value	PA total	*p* value
Stroop naming interference tendency	*r* = 0.185	0.214	*r* = −0.212	0.153	*r* = −0.22	0.129	*r* = −0.36	0.013 ^∗^
VLMT learning	*r* = 0.145	0.329	*r* = 0.188	0.207	*r* = 0.124	0.406	*r* = 0.239	0.106
VLMT consolidation	*r* = −0.338	0.020 ^∗^	*r* = −0.32	0.029 ^∗^	*r* = 0.446	0.002 ^∗∗^	*r* = −0.247	0.095
VLMT retrieval	*r* = 0.178	0.231	*r* = 0.198	0.182	*r* = 0.222	0.134	*r* = 0.377	0.009 ^∗∗^
TMT‐B	*r* = −0.281	0.056	*r* = −0.253	0.086	*r* = −0.315	0.031 ^∗^	*r* = −0.203	0.17
CORSI	*r* = −0.079	0.598	*r* = −0.014	0.923	*r* = 0.185	0.214	*r* = 0.055	0.715

*Note:* Asterisks indicate statistical significance levels:*p <0.05 denotes significance, and **p < 0.01 denotes high significance.

Sedentary time did not correlate with cognition.

#### 3.2.3.2. Cognition and HbA_1c_

Individual HbA_1c_ (%) correlates positively with the reaction time for CIN in the Stroop test (*r* = 0.296, *p* = 0.044) and negatively with the total verbal learning score in VLMT (*r* = 0.289, *p* = 0.049).

## 4. Discussion

The data analysis of the present study shows, for the first time, a significant association between physiological markers of aerobic fitness—both submaximal and maximal (ventilatory and metabolic)—overall levels of PA and cognitive performance in people with T2DM. A notable observation was that among individuals with T2DM, those with lower CRF and insufficient PA performed significantly worse overall in the cognitive domains examined. Diminished cognitive performance involves different domains such as executive function, processing speed, visuospatial processing and verbal memory. Last findings are in line with the meta‐analysis about cognitive dysfunction by individuals with T2DM, supporting diabetes‐associated performance decrements in these domains [5].

The study′s cross‐sectional design limits causal inference. Still, its findings are consistent with those of longitudinal and interventional research, reinforcing the role of modifiable lifestyle factors in preserving cognitive health in patients with T2DM [10–12]. Interestingly, higher CRF markers and PA levels showed a similarly significant association with cognitive performance, suggesting that improved CRF and PA can be equally beneficial for maintaining cognitive function in individuals with T2DM and in the nondiabetic population. However, the results reported here, indicating that compared with control subjects, people with T2DM were at least two to three times more likely to have cognitive performance 1 SD below the average, suggest that T2DM may have additional advantages to compensate for losses in neural efficiency and age‐related decrements in cognitive processes.

**Figure 4:**
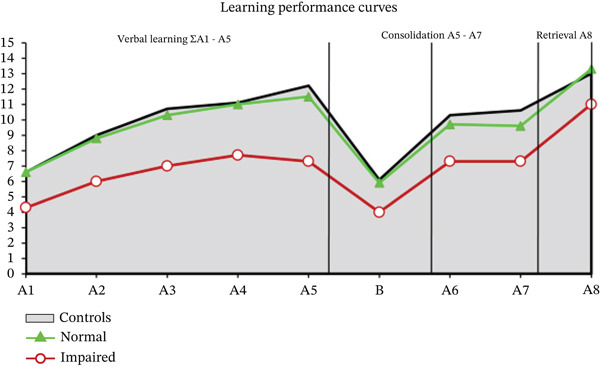
Subject′s learning and memory performance through the VLMT test. The curves show the number of words recognised correctly. A1–A5: items immediately remembered after presentations of 15 words (List A). B: recall of a presented interference list (list B). A6: early recall of List A, after this interference. A7: after a 30‐min break, free recall of List A. A8: words recognised from a larger list of verbally presented words after the delay. Background data: subjects with normal performance in the control group. Data of individuals with Type 2 diabetes: normal performance (green line), impaired performance (red line). For clarity, data of subjects with impaired performance in the control group are omitted from the diagram.

The proportion of recalled items (see VLMT learning performance curves, Figure 4), evidence that information is vulnerable to forgetting in all subjects, to an expected amount. However, analysis of memory performance revealed greater susceptibility to forgetting in individuals with lower aerobic capacity. In people with T2DM, submaximal endurance markers (Pow4La and *V̇*O_2_4La) as well as *V̇*O_2_peak showed a moderate negative correlation with consolidation of items to be learned (higher fitness values were associated with less loss of items, indicating better performance) (Figure 3c–e). Higher HRmax during exercise and greater total PA per week were also associated with better memory consolidation, consistent with Barnes et al. [24] who reported that higher *V̇*O_2_peak preserves cognitive function over a 6‐year follow‐up in adults over 55 years.

An important finding related to executive control functions. Our data show a significant correlation between CRF/PA levels and tasks requiring considerable inhibition and cognitive flexibility (aspects of executive function). Here, individuals with T2DM who achieve higher levels of PA, higher Pow4La and higher *V̇*O_2_peak also had significantly lower CIN tendency (naming performance is not much affected by interference, meaning stronger inhibitory function; Figure 3a), and greater cognitive flexibility (faster processing speed of sequential but switched tracing of numbers and letters; Figure 3b), respectively. Consistent with our study, Besnier et al. (2023) showed that, compared with diabetes‐free subjects, older adults (mostly men) with T2DM have lower *V̇*O_2_max and poorer executive function performance [25]. Previous longitudinal studies have also demonstrated that improvements in CRF enhance executive control in the elderly [26, 27] or in late middle adulthood [8]. Taken together, these findings suggest that high CRF/sufficient PA levels positively support executive function even in people with T2DM. Executive function [28] is essential for diabetes self‐care (e.g., monitoring blood glucose, making lifestyle decisions, inhibiting impulsive behaviours) and adequate disease management should be reflected in satisfactory metabolic control.

In addition, we found a relationship between executive function, learning and glycemic control in people with T2DM. Higher HbA_1c_ values were related to longer response times on tasks requiring inhibition of automatic responses (Stroop), indicating lower executive functioning. Similarly, higher HbA_1c_ levels were associated with fewer correct responses after five learning trials (VLMT), reflecting reduced learning performance. These results are consistent with previous reports linking poorer metabolic control to memory decrements [16].

Statistical analyses revealed no significant associations between cognitive task performance and blood pressure (at rest or under load), cholesterol, triglycerides, BMI, age or duration of diabetes. However, a potential causal association with other variables cannot be excluded due to the cross‐sectional design of the study, as well as the number and complexity of other possible risk factors. Possible known risk factors for cognitive decrements in people with T2DM include not only diabetes‐specific variables such as hyperglycaemia, hypoglycaemia, endothelial dysfunction, inflammation and both microvascular and macrovascular complications, but also disease‐independent factors, including premorbid cognitive status (i.e., impaired cognitive function at baseline might lead to reduced health care resulting in diabetes).

Healthy lifestyle behaviours, particularly habitual PA, are associated with better cognitive performance across populations [4, 29]. The cognitive benefits of exercise extend beyond improved cerebral blood flow and oxygen delivery [30, 31], including modulation of neurotransmitter systems related to memory [32]. PA influences multiple cortical and subcortical networks, as demonstrated by modern longitudinal neuroimaging studies in healthy adults [8, 33–36]. At the cellular/molecular levels, animal studies show that exercise increases the expression of neurotrophic factors, promotes angiogenesis, synaptogenesis and neurogenesis, thereby enhancing hippocampal neuroplasticity [37, 38]. In humans, exercise targets particularly BDNF, IGF‐1 and VEGF [39, 40]. Animal models of diabetes demonstrate exercise‐induced cognitive improvements via hippocampal BDNF upregulation [41–43], suggesting similar mechanisms may operate in individuals with T2DM.

Importantly, none of the psychiatric questionnaires (BDI, PANAS and STAI State) yielded clinically significant scores, indicating that the observed cognitive decrements were not secondary to depression or anxiety. Although STAI‐Trait scores were significantly higher in controls, they did not correlate with cognitive task performance.

### 4.1. Study Limitations

Given the use of a selected battery of neuropsychological tests and the subtle nature of cognitive changes, it is difficult to determine at the individual level whether a patient is affected based solely on test results.

Due to the exploratory nature of the study, the limited number of cognitively impaired individuals without diabetes limited the interpretability of the findings in this subgroup. The lack of formal adjustment for multiple comparisons may increase the risk of a Type I error; therefore, the findings should be interpreted with caution.

## 5. Conclusion

From our study, we can conclude that individuals with T2DM who report higher levels of PA or perform better in individual exercise testing exhibit improved cognitive functioning compared with their less active counterparts. This suggests that lifestyle choices, such as regular exercise, may make a difference and could help mitigate cognitive decline in vulnerable individuals. Regular PA may play a crucial role not only in enhancing CRF but also in reducing the adverse effects of modifiable risk factors that influence vascular and metabolic conditions. Furthermore, exercise is associated with processes likely to enhance cognition, such as angiogenesis, synaptogenesis and neurogenesis. These mechanisms may counteract existing or ongoing brain damage and delay the onset or progression of cognitive impairment. Although we cannot definitively state that CRF causes these effects in our cohort, the data contribute to the ongoing discussion of how best to support brain health in individuals with T2DM. Additionally, cost reductions may be achieved by reducing clinical deterioration and adverse outcomes across more patients.

Future randomised controlled trials and large cohort studies are needed to investigate the impact of supervised, individualised and age‐appropriate endurance training on markers of CRF and cognitive outcomes in individuals with T2DM.

## Author Contributions

Conception and design of the CODEX study were provided by Sandra Rojas Vega. Data acquisition was performed by Veronika Wahrmann, Sandra Rojas Vega, Lukas Scheef and Daniel Acero‐Moreno. Data analysis and interpretation were carried out by Andrea Solera‐Herrera, Sandra Rojas Vega and Ramin Vafa. Sandra Rojas Vega drafted and edited the manuscript. Lukas Scheef revised the article critically for important intellectual content.

## Funding

This research was supported by the internal funding of the German Sport University Cologne (GSU HIF Grant 920121).

## Disclosure

The funders of the study had no role in study design, data collection, data analysis, data interpretation, or writing of the report. The authors declare that there are no other relationships or activities that might bias or be perceived to bias their work. All authors read and approved the final manuscript.

## Conflicts of Interest

The authors declare no conflicts of interest.

## Data Availability

The data that support the findings of this study are not publicly available due to the information they contain that could compromise participant privacy and consent. The aggregate datasets are available from the corresponding author on reasonable request.
